# The indirect effect of physical activity on aggression among university student-athletes: the mediating role of state anger

**DOI:** 10.3389/fpsyg.2026.1717868

**Published:** 2026-02-27

**Authors:** Sevim Handan Yılmaz, Mert Ayrancı, Murat Sarıkabak, Mehmet Kemal Aydın, Metin Kuş, Abdulmenaf Korkutata, Murat Çelebi, Mehmet Civan

**Affiliations:** 1 Faculty of Sport Sciences, Bartın University, Bartın, Türkiye; 2 Faculty of Sport Sciences, Hitit University, Çorum, Türkiye; 3 Distance Education Application and Research Center, Hitit University, Çorum, Türkiye; 4 Faculty of Sport Sciences, Çanakkale Onsekiz Mart University, Çanakkale, Türkiye

**Keywords:** aggression, mediation analysis, physical activity, sports psychology, state anger, university student-athletes

## Abstract

**Introduction:**

This study examined whether physical activity (PA) is indirectly related to aggression through state anger among university student-athletes. Grounded in contemporary models of emotion regulation and aggression in sport, the study aimed to address inconsistencies in the literature regarding the potential effects of PA on aggressive tendencies.

**Methods:**

A cross-sectional survey design was employed with 284 student-athletes, and a mediation model (PROCESS Model 4) was used to explore the indirect pathway from PA to aggression via state anger.

**Results:**

While no significant direct effect was found between PA and aggression, PA was positively associated with state anger, which in turn was related to higher levels of aggression. The indirect effect of PA on aggression through state anger was statistically significant, suggesting that emotional responses may play a meaningful role in the relationship between PA and aggression.

**Discussion:**

These results may contribute to the sport psychology literature by offering insights into the emotional mechanisms underlying aggression in physically active populations and highlighting the potential value of monitoring situational anger in training contexts. From a practical perspective, the findings point to the potential benefit of integrating psychological skills training and anger regulation strategies into sport programs to help manage aggressive tendencies among university student athletes.

## Introduction

The increasing diversity of risk factors affecting mental health has led to growing interest in natural intervention strategies that can enhance individuals’ psychological resilience. In this context, PA is often regarded as a helpful approach that may support both physiological functioning and emotional regulation. An increasing number of studies have shown that regular PA reduces stress levels, improves mood, and supports overall psychological well-being (Herbert, 2022; Chan et al., 2023; Ren and Xiao, 2023). The psychological effects of exercise are explained through its influence on the nervous system and neurochemical balances, with particular emphasis on the role of serotonin, dopamine, and endorphin release in promoting positive mood states (Gorrell et al., 2022; Hossain et al., 2024).

However, the behavioral consequences of PA are not universally positive. In high-intensity, competitive, or emotionally demanding environments, PA may increase physiological arousal and stress reactivity, potentially triggering anger and aggressive responses (Jekauc et al., 2021; Piepiora et al., 2025). Sports environments can serve as both arenas for physical performance and settings of emotionally intense interactions, were competitive pressure, the desire to win, physical fatigue, and fear of failure can disrupt emotional balance and create a basis for aggressive reactions (Smith, 1990; Low et al., 2023). Thus, the effect of PA on aggression appears to be dependent on context and shaped by how individuals experience and manage specific emotional states during and after activity.

Recent studies have highlighted the psychological vulnerabilities of student-athletes, particularly in relation to aggression and emotional regulation. For example, Hagan (2021) found that anger appeared to be influenced by religious coping strategies among elite student-athletes, suggesting that state anger plays a key role in athletes’ emotional responses prior to competition. Similarly, Reza (2012) reported that aggression levels among student-athletes varied across different sports but were strongly associated with psychosocial and situational factors than with the sport itself. Moreover, Madden et al. (2021) showed that more than one-third of collegiate athletes were at high risk for anger, indicating that this transient emotional state may mediate the emergence of aggressive behaviors. These findings suggest the relevance of examining state anger as a potential emotional mechanism linking PA to aggression, especially within university student-athlete populations.

Within this framework, state anger may represent an important construct for understanding the link between PA and aggression. State anger refers to a transient, situationally induced emotional state characterized by feelings of tension, irritation and anger experienced at a particular moment. It is sensitive to immediate environmental cues and physiological arousal (Spielberger et al., 1999). PA has the potential to reduce this state anger through its calming effects on the nervous system; however, under certain conditions such as high training loads, competitive stress or perceived failure it may also lead to short-term increases in state anger (Shafir, 2015; Basso and Suzuki, 2016) For example, elevated catecholamine levels and heightened sympathetic activation following intense exercise can temporarily intensify feelings of in some individuals (Gavrilović et al., 2020). These findings suggest that the relationship between PA and state anger is complex and cannot be explained through a simple linear or uniformly beneficial pathway.

To conceptualize these processes, aggression research has drawn on well-established theoretical frameworks such as the General Aggression Model (GAM) and Berkowitz’s Cognitive Neo-Association Theory. GAM proposes that situational inputs such as physical exertion, competitive stress and contextual cues affect internal states involving cognition, arousal and emotion, which in turn shape appraisal processes and aggressive behavioral outcomes (Anderson and Bushman, 2002; Allen et al., 2018). Within this model, state anger is conceptualized as a proximal emotional state that can mediate the impact of situational stressors on aggressive behavior. Likewise, Cognitive Neo-Association Theory argues that negative affect and aversive arousal automatically activate associative networks related to anger and aggression, thereby increasing the likelihood of aggressive responses under certain conditions (Berkowitz, 1990). Recent sport-specific research further supports these assumptions by showing that anger and related emotional processes are closely linked with aggressive behavior in competitive environments (Sofia and Cruz, 2017; Havadar et al., 2024). Taken together, these frameworks propose that aggression in sport may be influenced by isolation but is closely tied to short-term emotional fluctuations particularly state anger that arises in response to situational and physiological demands.

Despite these theoretical foundations, empirical findings on the relationship between PA and aggression remain inconsistent. Some recent meta-analyses and reviews show that physical exercise can significantly reduce aggressive behavior among children and adolescents (Yang et al., 2023; Zhao and Wang, 2025), suggesting an overall beneficial effect of PA on aggression-related outcomes. However, other studies report that in competitive or high-intensity sport contexts, aggression and anger may not decrease or may even rise especially when arousal, stress or negative affect are elevated (van der Sluys et al., 2024). A likely explanation for these inconsistent findings is that prior research seldom examines state anger as a mediating emotional mechanism that could clarify when PA leads to reductions in aggression and when it may exacerbate it. Moreover, evidence focusing specifically on university student-athletes a group exposed to substantial training load, competitive stress and frequent fluctuations in emotional states, remains scarce, highlighting the value of conducting studies that integrate theoretical frameworks with emotional mediators like state anger to better understand the complex relationship between PA and aggression.

In light of these gaps, the present study aims to examine whether PA influences aggression indirectly through state anger among university student-athletes. Although contemporary aggression theories such as the GAM and Cognitive Neo-Association Theory highlight the role of short-term emotional states in aggressive behavior, empirical research has seldom tested state anger as a mediator in this relationship. Accordingly, this study adopts a theoretically grounded mediation approach to clarify how PA may shape aggressive tendencies through momentary anger responses in athletic settings.

Based on theoretical framework and prior empirical considerations, four hypotheses were formulated. First, PA was expected to be positively associated with state anger (H1), as training intensity and physiological activation may increase momentary emotional arousal. Second, state anger was hypothesized to be positively associated with aggression (H2), reflecting its role as a proximal emotional predictor of aggressive tendencies. Third, a direct relationship between PA and aggression (H3) was examined, although no strong theoretical evidence suggested a significant direct association. Finally, it was hypothesized that state anger would mediate the relationship between PA and aggression among university student-athletes (H4), in line with the proposition that short-term emotional reactions are key mechanisms linking situational inputs to aggressive behavior.

## Materials and methods

### Research model

This research is a quantitative study based on a cross-sectional survey design and employs a mediation model to examine the effect of PA on aggression through state anger. In the study, PA was designated as the independent variable (*X*), state anger as the mediator (*M*), and aggression as the dependent variable (*Y*). The model was tested using the PROCESS macro version 4.1 developed by Hayes (2018), specifically employing Model 4 to evaluate the indirect effect of PA on aggression (Hayes, 2018).

This model is one of the modern statistical methods that allows for testing mediation effects and is widely used, particularly in the fields of psychology and health sciences (Preacher and Hayes, 2008). Mediation analysis examines both the direct effect of PA on aggression and how this relationship is indirectly shaped through state anger.

### Participants and ethics statement

The sample of the study consisted of 284 university student-athletes residing in various regions of Türkiye. Participants were recruited using a convenience sampling method based on voluntary participation. Among the participants, 162 were male (57.0%) and 122 were female (43.0%), with ages ranging from 18 to 48 years (*M* = 21.92). All participants were students enrolled in Faculties of Sport Sciences and were actively involved in sports through university teams, private sports clubs, and national teams at different competitive levels. The sample included athletes competing at regional, national and international levels. Participants represented a variety of sports disciplines, including football, kickboxing, wrestling, handball, volleyball and bocce among others. Data was collected during the 2024-2025 academic year using both online and face to face survey methods. Online questionnaires were administered via Google Forms, while face to face data collection was conducted in training and academic settings.

Prior to data collection, ethical approval was obtained from the Non-Interventional Research Ethics Committee. All participants were thoroughly informed about the purpose, scope, and confidentiality principles of the study and signed informed consent forms before taking part in the data collection process. The study adhered to the ethical guidelines outlined in the Declaration of Helsinki, and all methods were performed in accordance with the relevant guidelines and regulations. Before proceeding with the data analysis, the dataset was examined for missing data and outliers; such cases were excluded from the analysis to ensure statistical consistency and reliability.

*A priori* power analysis was performed using G*Power 3.1 to determine the adequacy of the sample size for linear regression analysis with two predictors (PA and state anger). Assuming a small-to-medium effect size (*f*^2^ = 0.10), an alpha level of 0.05 and power of 0.95, the analysis indicated that a minimum of 158 participants was required. With 284 participants, the study exceeded the required sample size, ensuring sufficient statistical power.

### Data collection tools

#### International physical activity questionnaire-short form (IPAQ-SF)

Originally developed by Booth (2000), this scale was adapted into Turkish in 2005 (Öztürk, 2005). The scale consists of 7 items assessing PA performed for at least 10 min during the past 7 days. One MET-minute is calculated by multiplying the duration of the activity (in minutes) by its corresponding MET value. At rest, an individual consumes approximately 3.5 mL of oxygen per kilogram of body weight per minute, which is equivalent to 1 MET. According to IPAQ data, vigorous PA corresponds to 8.0 METs, moderate PA to 4.0 METs, and walking at a normal pace (4–5 km/h) to 3.3 METs. For the current study, the Cronbach’s alpha coefficient was calculated as 0.70.

#### State anger scale

To measure levels of anger, the State-Trait Anger Scale (STAXI), developed by Spielberger et al. (1999), was used. In the present study, the state anger subscale consisting of 10 items was administered to assess the intensity of anger experienced at a particular moment. Items are rated on a 4-point Likert scale ranging from 1 (not all) to 4 (very much so). An example item from the scale is “I feel very angry.” Higher scores indicate higher levels of state anger. STAXI is a widely used instrument in psychological research and has demonstrated satisfactory psychometric properties across different populations. In the present study, the Cronbach’s alpha coefficient for the state anger scale was calculated as 0.75.

#### Aggression questionnaire

To assess levels of aggression, the Aggression Questionnaire (AQ) developed by Buss and Perry (1992) was utilized. The scale consists of 29 items grouped into four sub-dimensions: physical aggression (9 items), verbal aggression (5 items), anger (7 items), and hostility (8 items). Items are rated on a 5-point Likert scale ranging from 1(extremely uncharacteristic of me). Example items include “I can resort to violence if necessary to defend my rights” for physical aggression “I cannot refrain from arguing when people disagree with me” for verbal aggression “Sometimes I feel like a powder keg ready to explode” for anger and “When someone is very nice to me, I wonder what they want from me” for hostility. Several items are reverse-scored and higher total scores indicate higher levels of aggression. In the present study, the scale demonstrated high internal consistency with a Cronbach’s alpha coefficient of 0.88.

### Statistical analysis

The research data were analyzed using IBM SPSS Statistics 25.0 and the PROCESS macro version 4.1 (Hayes, 2018). First, descriptive statistics were calculated for the study variables, and normality tests (skewness and kurtosis values) were conducted to assess the distribution characteristics of the data. To examine the relationships between variables, Pearson correlation analysis was performed. Subsequently, a linear mediation analysis was conducted using Model 4, as proposed by Hayes (2018), to test the mediating role among the variables. In this model, PA was entered as the independent variable (*X*), state anger as the mediator (*M*), and aggression as the dependent variable (*Y*). The indirect effect was tested using the bootstrap method with 5,000 resamples and a 95% confidence interval (CI). Both the direct and indirect effects of the model were reported separately to assess the structural validity of the relationships among the variables.

To reduce the potential influence of non-normal distribution and improve interpretability, the PA variable (MET) was standardized using *Z-*scores prior to analysis. This approach minimizes the impact of outliers, allows for more stable estimation in regression-based models and is commonly recommended in mediation analyses, particularly when variables vary in scale or distribution (Hayes, 2018).

The assumption of normality was assessed using Shapiro–Wilk tests and skewness-kurtosis values. Results showed that the state anger and aggression variables were normally distributed (Shapiro–Wilk *p* > 0.05), whereas the PA variable did not meet the normality assumption (Shapiro–Wilk *p* < 0.001; skewness = 1.648; kurtosis = 3.361). However, due to the sufficiently large sample size (*N* = 284), parametric analyses were considered robust to this violation. Moreover, the mediation analysis was conducted using bootstrapping, which is a non-parametric resampling method and does not require the assumption of normality (Hayes, 2018). Therefore, all analyses were considered statistically appropriate and reliable.

## Results

### Descriptive statistics

Table 1 presents the descriptive statistics for the main study variables, including PA (standardized MET scores), state anger and aggression. The table reports mean, standard deviation and observed minimum and maximum values for each variable.

**Table 1:** Descriptive statistics of physical activity, state anger, and aggression.

Variables	*N*	Minimum	Maximum	Mean	Std. deviation
*Z*-score (PA)	284	−0.90168	4.78	0.00	1.00
State anger	284	1.20	4.50	2.89	0.55
Aggression	284	1.38	4.41	2.85	0.55

The mean score for state anger was 2.89 (SD = 0.55), and for aggression it was 2.85 (SD = 0.56), indicating moderate levels among the participants. The PA variable was standardized using *Z*-scores prior to analysis, resulting in a mean of 0 and standard deviation of 1. These results provide an overview of the distribution and variability of the key constructions included in the study.

Before conducting the mediation analysis, Pearson correlation coefficients were calculated to examine the bivariate relationships among PA, state anger, and aggression.

As shown in Table 2, state anger was positively correlated with both aggression and PA, while a weak but significant positive relationship was observed between PA and aggression.

**Table 2:** Pearson correlation coefficients between PA, state anger, and aggression.

Variables	1	2	3
1. State anger	—		
2. Aggression	0.153**	—	
3. Physical activity	0.195**	0.116*	—

Values represent Pearson correlation coefficients. *p < 0.05, **p < 0.01 (two-tailed).

Table 3 summarizes the results of the mediation analysis conducted to examine the direct and indirect effects of PA on state anger and aggression. Using Model 4, the analysis statistically evaluated the effect of PA on state anger, the effect of state anger on aggression, and the potential indirect pathways between these variables.

**Table 3:** Direct and indirect effects of PA on state anger and aggression.

Variables	*B*	SE	*β*	*t*	*p*	95% CI
Dependent variable (state anger)						
Constant	2.8940	0.032	—	89.736	0.000	[2.830, 2.957]
PA (Met)	0.1080	0.032	0.195	3.344	0.000	[0.044, 0.171]
Dependent variable (aggression)						
Constant	2.455	0.177	—	13.806	0.000	[2.105, 2.805]
PA (Met)	0.049	0.033	0.088	1.479	0.140	[−0.0163, 0.115]
State anger	0.137	0.060	0.136	2.270	0.023	[0.018, 0.256]
Total effect (*X* → *Y*)	0.064	0.033	0.115	1.946	0.052	[−0.000, 0.129]
Direct effect (*X* → *Y*)	0.049	0.033	0.088	1.479	0.140	[−0.016, 0.115]
Indirect effect (*X* → *M* → *Y*)	0.014	0.008	0.026	—	—	[0.001, 0.032]

Note: *X* = PA (MET), *M* = State anger, *Y* = Aggression.

PA, Physical activity.

According to the analysis results, the effect of PA on state anger was found to be significant and positive (*B* = 0.1080, *p* = 0.0009), indicating that as PA increases, individuals also exhibit higher levels of state anger. However, the direct effect of PA on aggression was not statistically significant (*B* = 0.0494, *p* = 0.1401), suggesting that PA alone does not directly influence aggression levels. On the other hand, the effect of state anger on aggression was significant (*B* = 0.1372, *p* = 0.0239), meaning that an increase in state anger is associated with a rise in aggression levels.

**Figure 1:**
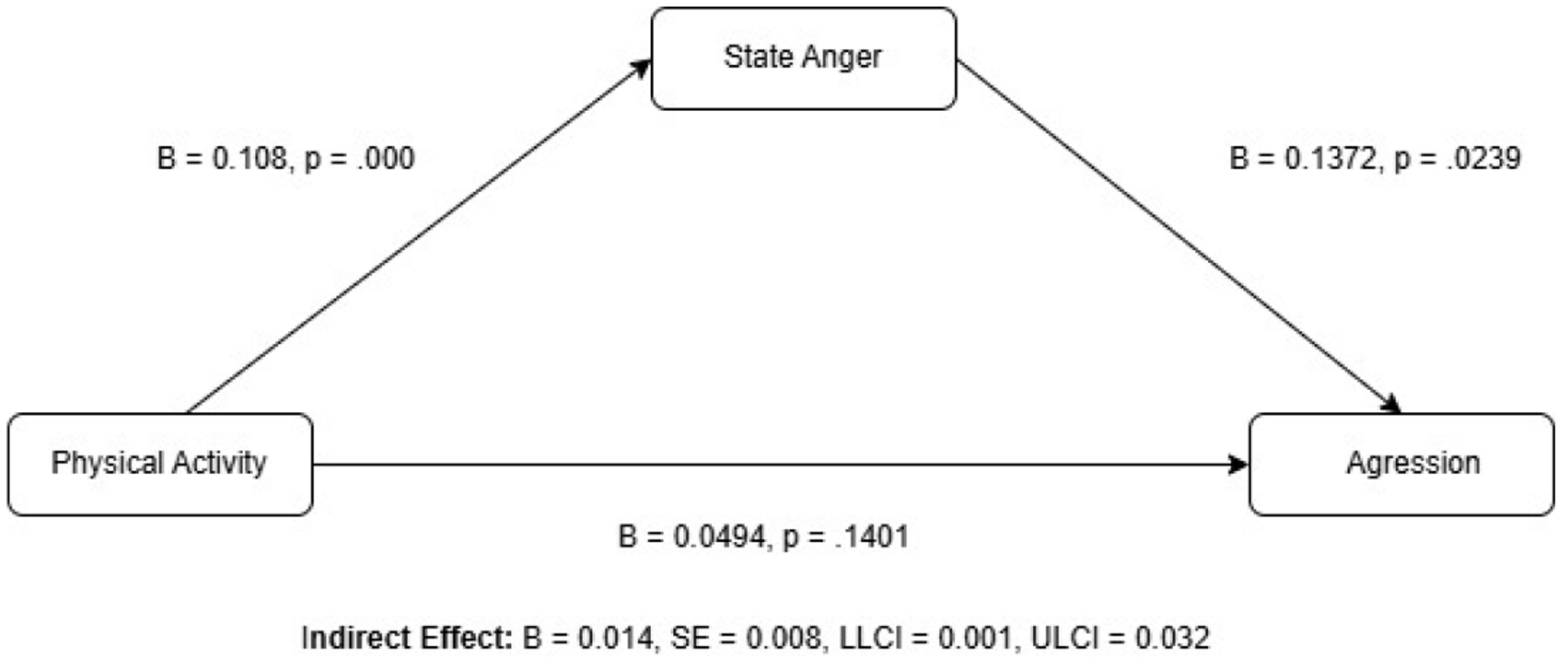
Mediating role of state anger in the relationship between PA and aggression.

Although the total effect of PA on aggression was marginally significant (*B* = 0.0643, *p* = 0.0526), the indirect effect through state anger was statistically significant (BootLLCI = 0.0015, BootULCI = 0.0325). These findings indicate that PA affects aggression not directly, but indirectly through state anger. In other words, as individuals engage more in PA, their levels of state anger increase, which in turn indirectly leads to higher levels of aggression. Figure 1 illustrates the mediating role of state anger in the relationship between PA and aggression.

## Discussion and conclusion

The present study examined whether PA influences aggression indirectly through state anger among university student-athletes, within a framework informed by the GAM and Cognitive Neo-Association Theory (Berkowitz, 1990; Anderson and Bushman, 2002; Allen et al., 2018). Consistent with this theoretical grounding, the results supported an indirect-only pattern: PA was positively associated with state anger (H1), state anger was positively associated with aggression (H2), the direct path from PA to aggression was non-significant (H3), and state anger significantly mediated the relationship between PA and aggression (H4). These findings suggest that, in this sample, aggression is not a simple behavioral consequence of being more physically active; rather, it emerges through short-term emotional processes activated during or following physical exertion.

With respect to H1, the positive association between PA and state anger indicates that higher levels of activity were linked to elevated momentary anger. This result is consistent with psychophysiological arousal and stress-related hormones such as cortisol, which can heighten irritability and negative affect in the short term (Raglin, 1992; Brownlee et al., 2005). In competitive or evaluative sport settings, such heightened arousal is often accompanied by situational stressors including performance pressure, ambiguous officiating decisions or interpersonal conflict with opponents and teammates (Nicholls et al., 2006; Weinberg and Gould, 2023). Under these conditions, transient increases in anger are not surprising and may reflect an immediate emotional response to perceived threat, frustration or injustice rather than a stable tendency toward aggression. At the same time, previous work has also shown that regular exercise can contribute to long-term anger regulation and reduced hostility when appropriately structured (Malhotre, 2019; Gökalp and Öztürk, 2021). Taken together, the current findings and prior evidence suggest that PA may simultaneously promote long-term emotional regulation while still producing short-term elevations in state anger during acute demanding situations.

The findings for H2 further reinforce the central role of state anger in aggression. In line with both General Aggression Model and the Cognitive Neo-Association perspective, state anger significantly predicted aggression, supporting the view that anger represents a proximal affective mechanism translating situational inputs into aggressive behavior (Berkowitz, 1990; Anderson and Bushman, 2002). Experimental research in social psychology shows that when state anger is experimentally induced by interpersonal insult, individuals display higher levels of aggressive responding toward the provocateur in laboratory paradigms, for example by administering more intense aversive stimuli (Harmon-Jones and Sigelman, 2001). Likewise, epidemiological evidence indicates that elevated state anger immediately prior to an event is associated with an increased risk of injury, particularly intentional injuries inflicted by others, suggesting that short-lived anger episodes can escalate into interpersonal violence in everyday life (Vinson and Arelli, 2006). Psychobiological work further demonstrates that state anger interacts with androgen levels to predict physical and verbal aggression in children, underlining the importance of transient emotional states as opposed to traits alone in the emergence of aggressive behavior (Sánchez-Martín et al., 2011).

Within sport contexts, studies using the State-Trait Anger Expression Inventory show that athletes in contact sports often report higher anger levels and more outward expression of anger than non-contact athletes or non-athletes, particularly in response to competitive stressors and interpersonal provocation (Robazza et al., 2006; Menéndez-Santurio and Fernández-Río, 2017). These findings indicate that intense, situational anger is a common part of athletes’ emotional experience and can be closely linked to behavior in highly charged competitive environments. Against this backdrop, the present study extended prior work by demonstrating that, among university student-athletes, state anger operates as a key proximal predictor of aggression, over and above PA itself. This supports the view that it is not PA per se, but the way athletes experience and regulate momentary anger in response to situational demands, that is critical for understanding aggressive tendencies in sport settings.

The lack of a significant direct effect of PA on aggression H3 adds an important nuance to the ongoing debate about how movement behaviors relate to aggressive outcomes. Recent systematic evidence suggests that this relationship is not linear: for example, a meta-analysis on bullying victimization, aggression, PA and sedentary behavior reported that aggression was more consistently associated with greater sedentary time, whereas victimization tended to co-occur with lower PA levels. In contrast, several studies indicate that when PA is embedded in structured or health-oriented contexts, it may help regulate emotions and reduce aggressive tendencies. Among Spanish undergraduates, higher PA and better emotional intelligence jointly predicted lower levels of violent behavior (Ubago-Jiménez et al., 2021). Longitudinal evidence from Chinese adolescents further shows that PA prospectively reduces aggression, partly through increases in self-control (Yue et al., 2025). Intervention work with violence-prone adolescents has likewise demonstrated that targeted PA and sports games can produce meaningful reductions in aggressive behavior over time (Alp and Ergül, 2018). At the same time, other findings point to a more differentiated pattern: PA has been found to be negatively related to proactive but not reactive aggression in children (Fite and Vitulano, 2011), and studies with sport science students during COVID-19 period report only limited differences in aggression sub-dimensions across daily activity levels (Şahinler et al., 2020). Taking together, these mixed results suggest that neither PA nor sport participation inevitably produces aggression. In line with this literature, the non-significant direct path observed in the present study indicates that, among trained university athletes, PA per se is not sufficient to elicit aggressive behavior; rather, the way athletes experience and regulate situational emotions such as state anger appears to be more critical for understanding aggression in sport settings.

This interpretation is reinforced when considering arousal-regulation frameworks such as the Inverted-U model, which posits that while moderate arousal may facilitate performance and emotional control, excessive arousal can impair cognitive regulation and increase impulsivity (Raglin, 1992; Weinberg and Gould, 2023). In sport settings, vigorous PA and competitive stress may elevate arousal but athletes who have undergone years of training may simultaneously develop coping strategies, emotional control and discipline that buffer against aggressive impulses. This balance likely reduces the likelihood that heightened state anger will be expressed as overt aggression, particularly in structured, rule-bound sport environments.

From a broader perspective, the mediated effect observed in this study aligns with research demonstrating that state anger can act as a mediator between stress and aggression in non-sport populations, where stressful experiences increase anger, which then elevates aggressive behavior (Sprague et al., 2011). By demonstrating similar mechanisms in an athletic context, this study contributes to the literature by showing that PA itself can function as a situational input through its physiological and competitive demands that influence aggression via state anger. Importantly, the present sample consists of university student-athletes rather than clinical or high-risk groups, suggesting that even in relatively well-adjusted populations, transient emotional states such as state anger warrant attention when assessing aggression-related risk.

The present study shows that the association between PA and aggression among university student-athletes is indirect, operating through state anger rather than a direct effect. PA predicted higher momentary anger and state anger subsequently predicted aggression, while no direct link between PA and aggression emerged. This pattern aligns with contemporary aggression theories that highlight proximal emotional processes and extends these insights to a sport-specific context.

Practically, the findings suggest that aggression management in athletic settings should emphasize monitoring and regulating state anger during training and competition, using strategies such as cognitive reframing, controlled breathing and coach-supported emotional feedback. In applied terms, coaches and sport psychology practitioners may benefit from implementing brief pre-competition anger check-ins to help athletes recognize elevated state anger before performance. In addition, teaching simple in-the-moment regulation techniques (e.g., short breathing resets or attentional refocusing cues) immediately following provocative events may reduce the likelihood that transient anger escalates into aggressive behavior.

A central contribution of this study is its focus on state anger, a transient, situational affect rather than trait anger, which dominates prior research. By highlighting how short-lived anger fluctuations during competition or intense physical exertion can trigger aggression, the study underscores that aggressive behavior is shaped not only by stable dispositions but also by the timing and intensity of situational emotional responses.

### Limitations and future research directions

This study has several limitations. First, due to its cross-sectional design, causal relationships between the variables cannot be directly established. Future research should consider using longitudinal and experimental designs to examine the long-term effects of PA on aggression. Second, the sample was limited to athlete students, which restricts the generalizability of the findings to the broader population. Testing similar models among individuals engaged in recreational sports and sedentary populations would be important to understand how these relationships may differ across contexts. In addition, gender-related differences were not examined in the present study, although previous research suggests that anger expression and aggressive tendencies may vary by gender. Furthermore, the study did not differentiate between contact and non-contact sports, which may involve different levels of physical confrontation and emotional demands and therefore may influence state anger and aggression in distinct ways.

Moreover, this study focused solely on state anger as a mediating mechanism. However, incorporating additional variables such as trait anger, emotion regulation skills, and motivation for sport participation could provide valuable insights in future research. Whether individuals engage in sport with a focus on competition or health may be a critical factor that shapes the impact of PA on aggression in different ways. Additionally, the competitive level of the student-athletes (e.g., international, national or regional/local level) was not systematically classified, even though athletes at different performance levels may experience varying degrees of competitive pressure and emotional intensity. Future studies should examine whether the proposed relationships differ according to athletes’ competitive level.

## Data Availability

The datasets supporting the findings of this study are publicly available on the Figshare platform and can be accessed via the following DOI: https://doi.org/10.6084/m9.figshare.28638692.
